# Therapeutic Potential of the ApoE-Mimicked Peptide COG133: Regulation of miRNA146‑a in Diabetic Fibroblasts and Antibacterial Activity

**DOI:** 10.1021/acsomega.5c10013

**Published:** 2025-12-19

**Authors:** Ayse Ak, Zehra Seda Halbutogullari, Ahu Soyocak, Ebru Onem, Rustu Tastan, Yusufhan Yazir

**Affiliations:** † Department of Medical Services and Techniques, Kocaeli Vocational School of Health Services, 52980 Kocaeli University, Kocaeli 41310, Turkey; ‡ Center for Stem Cell and Gene Therapies Research and Practice, Kocaeli University, Kocaeli 41001, Turkey; § Department of Medical Biology, Faculty of Medicine, 187981 Istanbul Aydin University, Istanbul 34295, Turkey; ∥ Department of Pharmaceutical Microbiology, Faculty of Pharmacy, 52994 Suleyman Demirel University, Isparta 32260, Turkey

## Abstract

COG133, an apolipoprotein E-derived mimetic peptide, has been proposed as a therapeutic candidate due to its immunomodulatory properties. Its potential role in diabetic wound healing, where impaired fibroblast function and chronic inflammation are major obstacles, remains largely unexplored. In this study, human diabetic dermal fibroblasts were treated with COG133 to evaluate its effects on cell viability, migration, and gene expression of ApoE, miR-146a, NF-κB, TRAF-6, and IL-6. In addition, the antibacterial and antibiofilm activities of COG133 were assessed against Gram-positive and Gram-negative bacteria. COG133 enhanced fibroblast migration without affecting viability, upregulated miR-146a, and reduced IL-6 and ApoE expression, while NF-κB and TRAF-6 remained unchanged. Antibacterial assays revealed inhibitory effects, with the lowest MIC against *Chromobacterium violaceum*, and a 55% reduction in *Pseudomonas aeruginosa* PAO1 biofilm formation. These results suggest that COG133 modulates inflammatory signaling and exhibits antibacterial properties, highlighting its therapeutic potential in supporting wound healing in diabetes.

## Graphical abstract

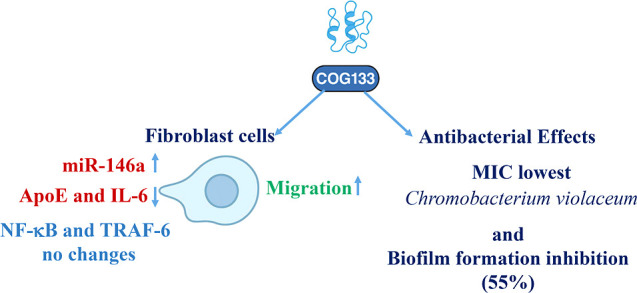

## 1 Introduction

Diabetes mellitus (DM) is a major global health concern, projected to affect 642 million people by 2040 according to the International Diabetes Federation (IDF). Type 2 diabetes (T2DM) is the most prevalent form, accounting for approximately 85–90% of all diabetes cases, and diabetes is responsible for 6.8% of global deaths in the 20–79 age group. A growing body of clinical evidence indicates that lifestyle modifications can effectively delay or prevent the progression of T2DM.^1^ Among the most devastating complications contributing to morbidity and mortality in diabetes are diabetic foot ulcers. In particular, nonhealing diabetic ulcers represent a major risk factor for infection, structural deformities, and lower extremity amputation. Alarmingly, the 5 year mortality rate among patients with diabetic ulcers exceeds that of breast cancer in women and prostate cancer in men. Patients with diabetes display impaired reparative responses across multiple stages of wound healing, including inflammation, angiogenesis, and re-epithelialization.^2^ In addition, genetic factors have been identified as important regulators in the development of T2DM and in determining patient responses to lifestyle interventions. This highlights the need for further research into molecular determinants underlying the etiology and complications of diabetes. In this regard, microRNAs (miRNAs) have attracted significant attention as key regulators of gene expression.^1^ Dysregulation of miRNAs can result in profound physiological abnormalities and chronic diseases such as diabetes. Notably, these molecules hold considerable promise as therapeutic targets for diabetic complications and as diagnostic biomarkers.^2,3^

MicroRNAs (miRNAs) are small noncoding RNAs of approximately 19–22 nucleotides that negatively regulate gene expression at the post-transcriptional level by binding to the 3′ untranslated regions of specific mRNAs. They are highly abundant in skin tissue and play essential roles in various biological processes, particularly in regulating both innate and adaptive immune responses.^4,5^ Among the key miRNAs involved in the inflammatory phase of wound healing is microRNA-146a (miR-146a), which serves as a crucial regulator of inflammatory and immune responses.^6^ It is induced by pro-inflammatory stimuli such as interleukin-1β (IL-1β) and tumor necrosis factor-α (TNF-α), and exerts its effects by targeting IL-1 receptor-associated kinase 1 (IRAK1) and TNF receptor-associated factor 6 (TRAF6). These adaptor molecules activate the nuclear factor kappa enhancer binding protein (NF-κB) pathway, thereby promoting the expression of pro-inflammatory cytokines including interleukin-6 (IL-6) and interleukin-8 (IL-8). Dysregulation of miR-146a has been implicated in several chronic inflammatory disorders such as psoriasis, rheumatoid arthritis, and systemic lupus erythematosus.^5,7^ Beyond these roles, miRNAs have been shown to contribute significantly to the pathophysiology of diabetes and its complications, and are considered promising therapeutic targets and diagnostic biomarkers.^8^ The biogenesis and activity of miR-146a are also modulated by apolipoprotein E (ApoE). ApoE has been shown to regulate chronic inflammation, particularly in atherosclerosis, by influencing both innate and adaptive immunity.^9^ Furthermore, studies suggest that ApoE can reduce tissue inflammation by limiting the release of cytokines such as transforming growth factor-β (TGF-β) and IL-6.^10,11^ Nevertheless, its precise immunoregulatory mechanisms remain incompletely understood.^9^ Only a limited number of studies have also linked ApoE to delayed wound healing.^12–14^ Inflammation is a central factor in impaired wound healing in diabetic patients, and miR-146a plays a pivotal role in maintaining immune balance through negative feedback regulation of the NF-κB pathway. Thus, fine-tuning of miR-146a expression may represent a promising therapeutic approach. Despite ApoE’s established role in inflammation and in miR-146a regulation, the effects of its mimetic peptide COG133 on diabetic fibroblasts remain unclear. Investigating the impact of the ApoE-mimetic peptide COG133 on miR-146a and inflammatory responses may therefore contribute to the development of novel therapeutic strategies to enhance wound healing in diabetic patients.

## 2 Materials and Methods

### 2.1 Cell Lines

Human diabetic dermal fibroblast cells (DDF) (catalog no.: HD2-6067) were cultured in 500 mL basal medium supplemented with 0.5 mL fibroblast growth factor, 0.5 mL hydrocortisone, 5.0 mL l-glutamine, 5.0 mL antibiotic-antimycotic solution, and 50 mL fetal bovine serum in a 5% CO_2_ incubator at 37 °C. COG133 peptide (catalog no. A1131 5MG APEX BIO), with a molecular weight of 2169.73 g/mol (C_97_H_181_N_37_O_19_), was dissolved in the medium at final concentrations of 0.2 μM, 1 μM, and 5 μM. The peptide was applied to the cells seeded at a density of 1 × 10^6^ cells/well and incubated for 72 h.

#### 2.1.1 Cell Viability

MTT (3-(4,5-dimethylthiazol-2-yl)-2,5-diphenyltetrazolium bromide) assay was conducted to assess the cytotoxic effects of the peptide on cell lines. Cells were seeded into 96-well microplates at a total medium volume of 200 μL per well. After seeding, cells were allowed to incubate for 24 h. Following the incubation period, the medium was removed from the wells, and cells were exposed to different concentrations of COG133 for 24, 48, and 72 h. At the end of each respective incubation period, the medium was aspirated and replaced with 200 μL of MTT solution, and the plates were further incubated at 37 °C in a 5% CO_2_ atmosphere for 3 h. After the incubation period, the MTT solution was removed, and 200 μL of dimethyl sulfoxide (DMSO) was added to dissolve the formazan crystals formed by MTT. Subsequently, absorbance was measured at 570 nm using an ELISA reader (Thermo Scientific, USA).

#### 2.1.2 Migration Assay

Tissue culture plates were marked with 15 straight lines using a glass marker, with 3 additional vertical lines intersecting these lines. Cells were initially seeded onto the plates, and after 24 h, using a 1 mL pipet tip, three wound models were created along the base of the plates following the 3 marked lines. Cells were treated with different concentrations of COG133 for 24, 48, and 72 h to study migration. The width of the wounds at the intersections of all lines was measured at 0, 24, 48, and 72 h. Subsequently, migration percentages were determined using the formula

$$migration\;percentage=\left(\frac{{migration}_{0}-{migration}_{time}}{{migration}_{0}}\right)\times 100$$

#### 2.1.3 Gene Expression Analysis

Total RNA was extracted from cells to evaluate the expression levels of NFκB1, TRAF6, IL-6, ApoE genes and miRNA-146a using the EcoPURE total RNA kit (EcoTech Biotechnology, cat. no.: E2075). RNA isolation was performed according to the manufacturer’s instructions. Briefly, chloroform was added to the lysate to induce phase separation by centrifugation, and the RNA-containing aqueous phase was mixed with ethanol before being applied to the purification column. DNA contamination was removed with DNase I provided in the kit. The concentration and purity of RNA were determined using a NanoDrop 2000c spectrophotometer (Thermo Scientific, USA), and samples with an A260/280 ratio between 1.8 and 2.0 were accepted as pure and stored at −80 °C until use.

cDNA synthesis for mRNA analysis was performed using the RT^2^ first strand kit (Qiagen, cat. no.: 330404), which includes a genomic DNA elimination step and optimized buffer systems. Reverse transcription was conducted according to the manufacturer’s instructions, with incubation at 42 °C for 15 min followed by enzyme inactivation at 95 °C for 5 min cDNA synthesis for miRNA analysis was carried out using the miRCURY LNA RT kit (Qiagen, cat. no.: 339340) under the manufacturer’s recommended conditions, involving incubation at 42 °C for 60 min and enzyme inactivation at 95 °C for 5 min. All cDNA samples were stored at −20 °C until further use.

Real-time PCR (qRT-PCR) analyses were performed using the Roche LightCycler 480 real-time PCR system (Roche Diagnostics, Germany). Reaction mixtures were prepared according to the manufacturer’s instructions using the RT^2^ SYBR Green FAST Mastermix (Qiagen, cat. no.: 330600), and gene-specific primer sets were added. The primer sequences for the target genes were as follows:

IL-6 (forward 5′-CACTCACCTCTTCAGAACGAAT-3′, reverse 5′-GCTGCTTTCACACATGTTACTC-3′), ApoE (forward 5′-GACAATCACTGAACGCCGAAG-3′, reverse 5′-TGCGTGAAACTTGGTGAATCTT-3′), NF-κB (forward 5′-GAGACATCCTTCCGCAAACT-3′, reverse 5′-GGTCCTTCCTGCCCATAATC-3′), and TRAF-6 (forward 5′-AAGGGATGCAGGTCACAAATGT-3′, reverse 5′-TTTTCCAGCAGTATTTCATTGTCAA-3′). GAPDH was used as the reference gene for normalization (forward 5′-ACCACAGTCCATGCCATCAC-3′, reverse 5′-TCCACCACCCTGTTGCTGTA-3′). Thermal cycling conditions were applied according to the manufacturer’s protocol, which included an initial activation at 95 °C for 10 min followed by 40 cycles of denaturation at 95 °C for 15 s and annealing/elongation at 60 °C for 1 min. Fluorescence signals recorded during each cycle were used to determine gene expression levels.

miRNA expression analyses were performed using the miRCURY LNA SYBR green PCR kit (Qiagen, cat. no.: 339345). Reaction mixtures were prepared according to the manufacturer’s instructions, and gene-specific primers were added. For the target, the hsa-miR-146a-5p primer set (Qiagen, GeneGlobe ID: YP00204688, cat. no.: 339306) was used, while SNORD44 (Qiagen, GeneGlobe ID: YP00203902, cat. no.: 339306) served as the reference. Thermal cycling conditions were applied in accordance with the manufacturer’s protocol, beginning with an initial heat activation at 95 °C for 2 min, followed by 40 cycles of denaturation at 95 °C for 10 s and a combined annealing/extension step at 56 °C for 60 s, during which fluorescence signals were collected to determine miRNA expression levels.

All experiments were performed in technical triplicates and with at least three biological replicates. No-template controls (NTC) and no-RT controls were included as negative controls. Relative gene expression levels were calculated using the 2^(−ΔΔCt)^ method.^15^

### 2.2 Antibacterial Activity

To investigate the antibacterial and antiquorum sensing effects of COG133, a total of six strains were included in the study: three Gram-positive [*Bacillus cereus* (ATCC 11778), *Enterococcus faecalis* (ATCC 29212), methicillin-resistant *Staphylococcus aureus* MRSA (ATCC 43300)] and three Gram-negative [*Chromobacterium violaceum* (ATCC 12472), *Pseudomonas aeruginosa* (ATCC 27853), *P. aeruginosa* PAO1].

#### 2.2.1 Determination of Minimum Inhibitory Concentration Values Using the Microdilution Method

The minimum inhibitory concentration (MIC) microdilution method was used to determine the antibacterial activity of COG133 on the bacteria to be used in the study. In this method, 96-well microplates were used, and COG133 dissolved in sterile water was added to the wells of Mueller Hinton broth medium in 2-fold serial dilutions. 5 μL of a bacterial suspension prepared according to a turbidity of 0.5 McFarland (10^8^/mL) was added to the microplates and incubated overnight at 30/37 °C. Following incubation, the microplates were evaluated, and the minimum inhibitory concentration (MIC) was determined, which was the lowest concentration at which no growth occurred.

#### 2.2.2 Biofilm Production

The effect of COG133 on biofilm formation by *P. aeruginosa* PAO1 was investigated using the crystal violet method.^16^ For this purpose, 16–18 h bacterial cultures were adjusted to a turbidity of 0.5 McFarland, Luria–Bertani broth (LBB) was added, and 20 μL of COG-133 at a nonantibacterial concentration (0.02 mM) was added and incubated at 37 °C for 48 h. Following incubation, the cultures in the plates were decanted and washed three times with sterile water. 125 μL of crystal violet (0.1%) was added to the wells to stain the biofilm layer for 15 min. At the end of this period, the dye was decanted, the wells were washed again with distilled water, and 200 μL of 95% ethanol was added. Absorbance values were read at 570 nm on an ELISA reader (Biotek Epoch 2 Miproplate Spectrophotometer) to evaluate the results. Only the *P. aeruginosa* PAO1 culture was used as a positive control.

### 2.3 Statistical Analyzes

The results obtained from the cells were evaluated using parametric and nonparametric statistical analysis methods based on the type and distribution of variables. A type I error probability of 0.05 was set for all analyses, and test results with *p*-values less than 0.05 were considered statistically significant. For the antibacterial activity experiments, randomized complete block design trials were conducted with three replications. The data obtained were subjected to analysis of variance using JMP 8 statistical software package. Statistical differences were annotated using the LSD multiple comparison test.

## 3 Results

### 3.1 Cell Viability Assay

The effects of COG133 at concentrations of 0.2 μM, 1 μM, and 5 μM on cell viability in DDF cells are shown in Figure 1. None of the applied concentrations caused a reduction in cell viability below 70%. A concentration of 1 μM COG133 was used for gene expression and migration assays.

**Figure 1:**
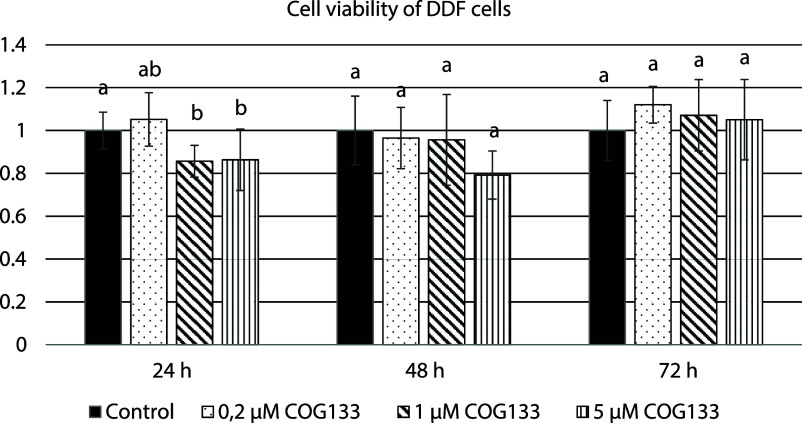
Effects of COG133 concentrations on cell viability in DDF cells (*n* = 8). Bars indicate the standard deviation (SD). Statistical differences between groups were determined by one-way ANOVA followed by Tukey’s post hoc test. Groups at each hour with different letters are statistically different (*p* < 0.05).

### 3.2 Migration Assay

Based on the data obtained from cell viability assays, a noncytotoxic concentration of 1 μM COG133 (less than 20%) was used in migration analyses. As shown in the Table 1 and Figure 2, after 72 h, cell migration was 87% in the control group, whereas it was 90% in the group treated with 1 μM COG133.

**Table 1:** Migration Percentage of Diabetic Dermal Fibroblasts at 24, 48, and 72 h (*n* = 3)

groups	24 h	48 h	72 h
DDF control	48%	67%	87%
DDF 1 μM COG133	39%	83%	90%

**Figure 2:**
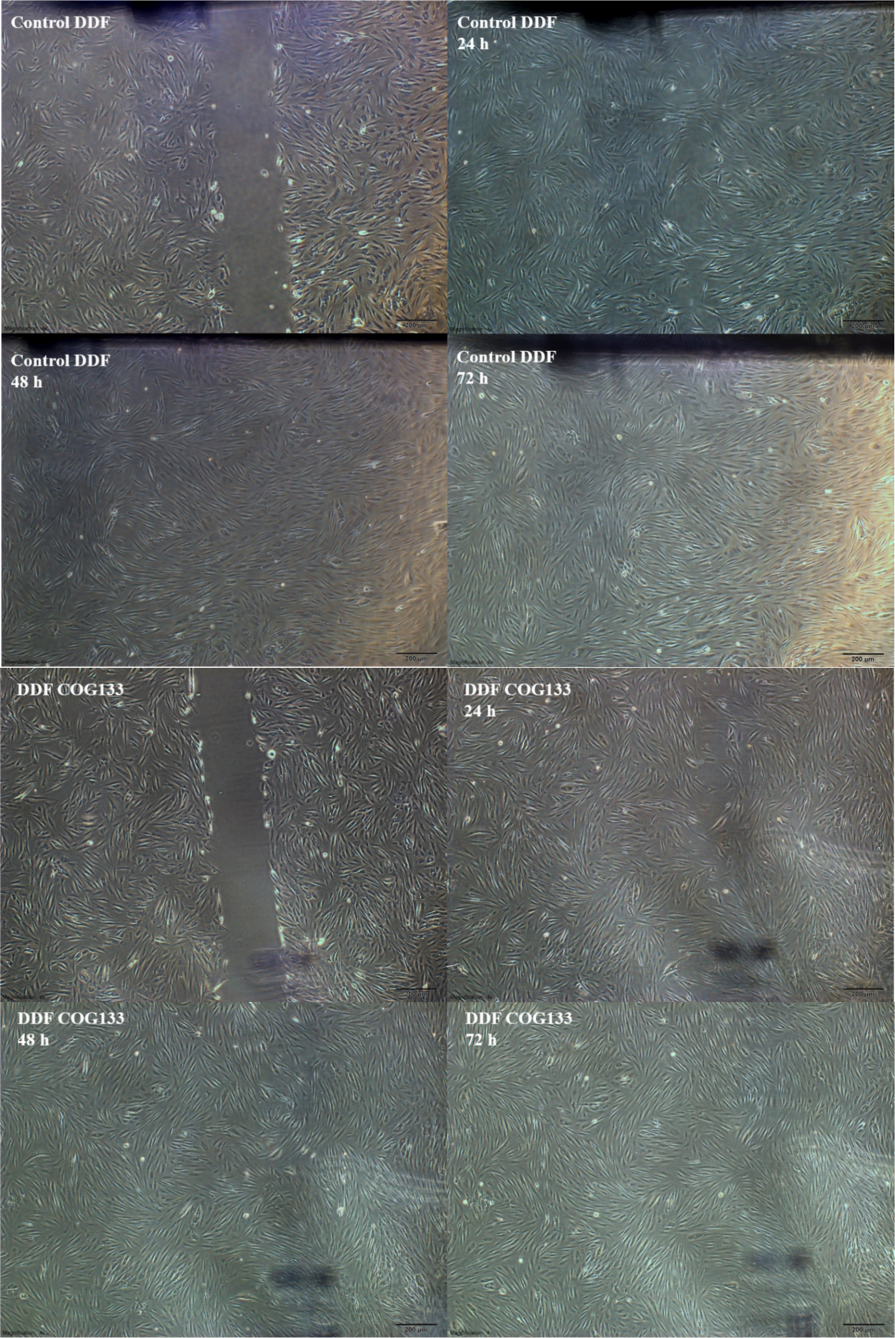
Migration assay images at 24, 48, and 72 h following treatment with 1 μM COG133 in diabetic dermal fibroblast cells.

### 3.3 Gene Expression Assays

After 72 h of 1 μM COG133 treatment, compared to the DDF control group, ApoE (*p* = 0.004) and IL-6 (*p* < 0.001) gene expressions were decreased in the treatment groups, while NF-κB (*p* = 0.232) and TRAF-6 (*p* = 0.217) gene expressions remained unchanged. In contrast, miR-146a expression (*p* < 0.001) was found to be increased (Figure 3).

**Figure 3:**
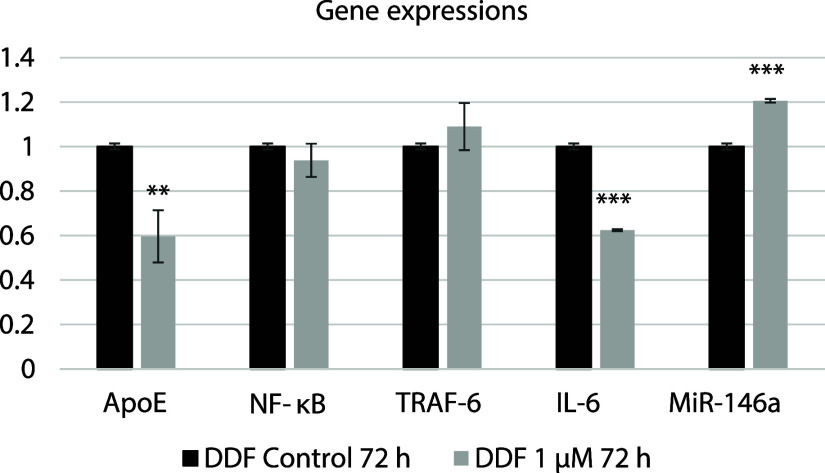
Gene expressions after COG133 treatment in DDF cells at 72 h. ** Differences between mean values followed by different letters of compounds are statistically significant (*p* < 0.01). *** Differences between mean values followed by different letters of compounds are statistically significant (*p* < 0.001).

### 3.4 Antibacterial Activity

MIC values for COG133, which demonstrate its antibacterial activity against some Gram-positive and Gram-negative bacteria, are given in Table 2. Based on the values, MIC values on Gram-negative bacteria were observed to be lower than those on Gram-positive bacteria. The lowest MIC concentration was observed on *C. violaceum* ATCC 12472 at a concentration of 3.125 μM.

**Table 2:** MIC Values of COG133 on the Studied Strains

strains	MIC value
P. aeruginosa PAO1	6.25 μM
P. aeruginosa ATCC 27853	12.5 μM
C. violaceum ATCC 12472	3.125 μM
S. aureus ATCC 25923	>20 μM
MRSA ATCC 43300	>20 μM
E. faecalis ATCC 29212	>20 μM

### 3.5 Antiquorum Sensing Activity

The inhibitory effect of COG133 on biofilm formation in *P. aeruginosa* PAO1 was investigated using the crystal violet test, a spectrophotometric method, and it was observed that 55% inhibition persisted and was significantly supported at a concentration of 2 μM (Figure 4).

**Figure 4:**
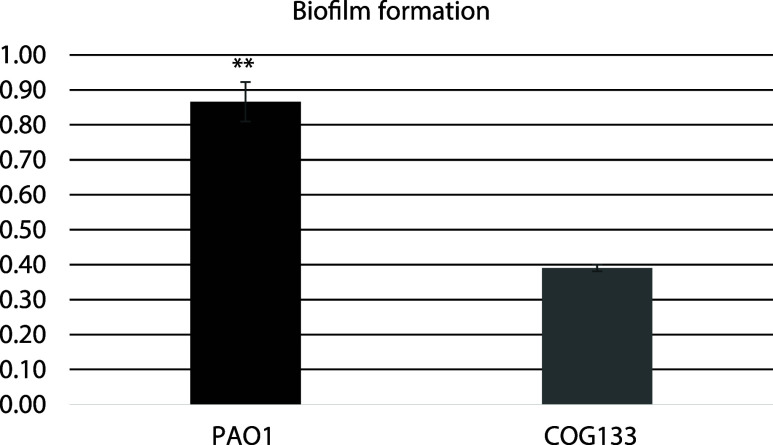
Inhibitory effect of COG133 on biofilm formation. ** Differences between mean values followed by different letters of compounds are statistically significant (*p* < 0.01).

## 4 Discussion

Administration of 1 μM ApoE mimetic COG133 to diabetic dermal fibroblast cells did not affect cell viability or migration after 72 h, while it decreased ApoE and IL-6 gene expression, increased miR-146a expression, and did not affect NF-κB and TRAF-6 gene expression. ApoE is an important protein not only in lipid metabolism but also in the regulation of immunity and inflammation. A significant part of this regulatory effect arises from its ability to control microRNA networks, particularly miR-146a. ApoE increases the expression of PU.1 (Spi1), a transcription factor that influences miR-146a. Therefore, the presence of ApoE in the cell promotes miR-146a production. A decrease in ApoE expression may also lead to reduced miR-146a transcription.^17^ In addition to its role in clearing atherogenic lipoprotein remnants from plasma, ApoE has been shown to regulate cellular signaling in immune cells and the vascular wall under the control of microRNAs. Studies conducted in mouse models have shown that ApoE influences NF-κB-mediated inflammatory processes and atherosclerosis by increasing miR-146a expression.^18,19^ In the study by Almzaiel et al. (2021), low ApoE levels were shown to contribute to a decrease in miR-146a levels and suppression of cellular activation. These studies highlight that miR-146a is regulated by ApoE at the cellular level and may represent a potential target in the resolution of inflammation and atherosclerosis.^18^ Consistent with these findings, our study demonstrated that the ApoE mimetic protein COG133 was able to increase miR-146a expression. Although ApoE expression levels were low in ApoE knockout mice, the apolipoprotein A-I mimetic peptide administered remained functional.^20^ Studies performed with the peptide 4F demonstrated that it significantly increased ApoE secretion and lipidation; however, this effect occurred without changes in gene transcription or translation, instead resulting from the functional activation of intracellular ApoE protein.^21,22^ In our study, it was also observed that COG133 could inhibit ApoE mRNA expression while increasing miR-146a expression. In our study, while the ApoE mimetic peptide increased miR-146a expression, it did not significantly alter TRAF-6 and NF-κB mRNA levels. miR-146a is an important microRNA that regulates inflammation by inhibiting the NF-κB pathway.^23,24^ However, in periodontitis, despite overexpression of miR-146a, increases in IL-1β and TNF-α have been observed, indicating that its effect alone may be insufficient. This apparent contradiction can be explained by the complexity of inflammatory signaling networks. NF-κB activation is not solely dependent on miR-146a; it can also be triggered by various stimuli such as bacterial lipopolysaccharides (LPS), Toll-like receptors (TLR2 and TLR4), and other proinflammatory pathways. Therefore, inflammation is a complex network involving the interaction of numerous molecules and pathways, making it difficult to isolate the effect of a single factor.^23^ Moreover, contradictory information exists regarding the role of ApoE in inflammation through NF-κB. The ApoE4 isoform can activate the NF-κB and MMP-9 signaling pathways more strongly, leading to blood–brain barrier disruption and increased cerebral edema. In contrast, the ApoE3 isoform suppresses this pathway, reducing these detrimental effects and exerting neuroprotective roles.^25,26^ Similarly, although the anti-inflammatory role of miR-146a has been demonstrated in models of Alzheimer’s disease, intracerebral hemorrhage, spinal cord injury, amyotrophic lateral sclerosis, and traumatic brain injury, some studies have shown that increased miR-146a expression may exacerbate damage in models of stroke, ischemia-reperfusion injury, and neuropathic pain. These contradictions may arise from disease-type differences, miRNA regulatory mechanisms, interactions between pro- and anti-inflammatory pathways, or differential expression patterns.^27^ Because inflammatory responses cannot be fully explained by a single molecule or pathway, and alternative signaling cascades may contribute to the persistence of inflammation, the development of multitarget therapeutic strategies is required. In this context, comprehensive studies employing various animal models and clinical research are needed to further elucidate the therapeutic potential of the miR-146a and ApoE axis.^27^ Therefore, it can be suggested that increasing miR-146a expression alone by the ApoE mimetic peptide may not fully reflect its effects on NF-κB and TRAF-6 gene expression. Although the anti-inflammatory effect of miR-146a has been reported in numerous studies, this negative feedback loop has not been demonstrated in some experimental settings.^28–31^ The expression level and suppressive effects of miR-146a may vary in a tissue-specific manner. In diabetic rat kidney cells, a 3-fold increase in miR-146a expression was observed, but no significant decrease in IRAK1 and TRAF6 was detected. This may indicate that IRAK1 and TRAF6 are regulated at other control levels or may respond differently to miR-146a depending on cytokine stimuli.^29^ In peripheral mononuclear cells of rheumatoid arthritis (RA) patients, miR-146a was found to be overexpressed, yet no change in IRAK1 and TRAF6 expression was observed. In systemic lupus erythematosus (SLE), miR-146a expression was decreased, while IRAK1 and TRAF6 expression remained unchanged. It has been suggested that although TRAF6 may be under the regulatory influence of miR-146a, their simultaneous expression levels may be independent and differ from one another. Similarly, regulation of IRAK1 and IRAK2 by miR-146a and NF-κB may differ in human astrocytes under stress and in Alzheimer’s disease.^30^ Since inflammatory pathways can be modulated by numerous factors, the degree of upregulation of miR-146a as a negative regulator is highly limited and may be insufficient to suppress the inflammatory response.^31^ In our study, although COG133 administered at 1 μM increased miR-146a expression in diabetic dermal fibroblast cells compared with the control group, it did not cause significant changes in TRAF-6 and NF-κB mRNA levels. The ApoE [133–149] peptide has been shown to significantly reduce serum TNF-α and IL-6 production at both mRNA and protein levels in mice.^32,33^ Moreover, increased expression of miR-146a has been shown to reduce levels of cytokines such as TNF-α, IL-1β, and IL-6.^34^ As in our study, the ApoE mimetic COG133 may suppress IL-6 expression via miR-146a or independently.

In this study, the antibacterial effect of COG133, one of the few synthetic peptides mimicking ApoE, was examined on several Gram-positive and Gram-negative bacteria, and the lowest effective concentration was found to be 3.125 μM against the Gram-negative *C. violaceum* ATCC 12472. No similar study was found in the current literature. In a study using ApoE derivatives rApoE PM (133–150), sApoE (133–150), Ac-ApoE (133–150)-NH2, and COG133 r­(P)­GKY20, antibacterial effects against Gram-positive and Gram-negative bacteria were evaluated, and MIC values were reported to range from 3.12 μM to >100 μM.^35^ In addition, native ApoE protein has been found to display antibacterial effects, especially against Gram-negative bacteria such as *Escherichia coli*.^36^ Antimicrobial peptides exhibit a broad spectrum of activity ranging from antibacterial effects against Gram-positive and Gram-negative bacteria to specific antifungal activity. Although they have MIC values comparable to classical antibiotics, they have been shown to exhibit more rapid bactericidal effects at these MIC values and to prevent the development of multidrug-resistant strains. Furthermore, they are known to act synergistically with conventional antibiotics, enhancing their effectiveness.^37,38^ *P. aeruginosa* is an opportunistic pathogen, and biofilm formation plays a critical role in the pathogenesis of the infections it causes, leading to increased morbidity and mortality, particularly in hospital-acquired infections. Bacterial biofilms are responsible for many infections, including dental caries, osteomyelitis, and cystic fibrosis. While acute infections caused by planktonic bacteria can be treated with antibiotics, infections caused by biofilm-forming bacteria are difficult to treat and can become chronic.^39^ In this study, the inhibitory effect of COG133 on biofilm formation by *P. aeruginosa* PAO1 was found to be 55% at a sub-MIC concentration (2 μM), and this effect was statistically significant. The human cathelicidin LL-37, a well-known antibiofilm agent, inhibits *P. aeruginosa* biofilm formation at a concentration of 0.5 μg/mL by acting on the quorum sensing system and preventing bacterial adhesion to surfaces.^40^ The antibiofilm activity of COG133 at concentrations below 2 μM suggests that it may be highly valuable in preventing biofilm formation, which is a major challenge in combating bacterial infections, and may be effective in the development of antibiofilm agents.

In addition to COG133, several other peptides have shown promising potential in diabetic wound healing. CyRL-QN15, an ultrashort cyclic peptide, has been suggested by in vivo studies to function as a Toll-like receptor 4 (TLR4) antagonist and to bind the Frizzled-7 receptor. In vitro models have further demonstrated that it can modulate stem cell functions through the Frizzled 8 (FZD8)/β-catenin axis, thereby accelerating skin wound regeneration. Consequently, it enhances dermal repair, supports hair follicle regeneration, and improves diabetic wound healing in preclinical models.^41–43^ Composite hydrogels incorporating this peptide have also been developed to promote the healing of infected wounds, while simultaneously enhancing antibacterial activity.^44^ Another promising therapeutic candidate is FZ1, a cyclic heptapeptide identified as the first peptide agonist targeting integrin αvβ3, which has demonstrated significant pro-healing effects in diabetic wounds.^45^ Andersonin-W1 represents an additional peptide with antibacterial properties. It can stimulate keratinocyte proliferation, migration, and scratch repair, promote macrophage proliferation, and induce tube formation in HUVECs via the TLR4/NF-κB molecular axis, ultimately accelerating the healing of diabetic wounds.^46^

In conclusion, the ApoE mimetic peptide COG133 possesses multifunctional biological properties due to its ability to modulate inflammatory signaling and exhibit antimicrobial activity. However, the in vitro models used in this study have certain limitations. While inflammation in diabetic wound models is already a complex process involving multiple signaling pathways, in vivo conditions further increase this complexity due to cell–cell interactions, immune responses, and microenvironmental factors. Although the concentration of COG133 used in our study and the 72 h treatment increased miR-146a expression, it may have been insufficient to activate TRAF-6 and NF-κB signaling. Additionally, the TRAF-6/NF-κB signaling pathway can be regulated by various biological processes. Therefore, advanced in vitro, ex vivo, and in vivo studies evaluating higher concentrations and longer treatment durations are required to more comprehensively elucidate both the pathways involved in inflammation and the translational aspects. The ApoE mimetic COG133 may represent a valuable therapeutic molecule with both anti-inflammatory and antimicrobial properties.
